# Cancer-related illness perceptions and causal attributions among adolescents and young adults with cancer: associations with health-related quality of life

**DOI:** 10.1007/s11764-025-01913-2

**Published:** 2025-10-27

**Authors:** Katie Darabos, Shannon Desbiens, Sean McHugh

**Affiliations:** 1 Rutgers School of Public Health, 683 Hoes Lane West, Piscataway, NJ 08854 USA; 2 Rutgers Cancer Institute, 195 Little Albany Street, New Brunswick, NJ 08901 USA

**Keywords:** Adolescent and young adults, Causal attributions, Health-related quality of life, Illness perceptions

## Abstract

**Purpose:**

Nearly 90,000 adolescents and young adults (AYAs) are diagnosed with cancer each year in the United States. The developmental “off-time” nature of cancer during adolescence and young adulthood is associated with significant distress. The ways in which AYAs formulate beliefs about cancer (e.g., how much cancer affects their lives and causal attributions) may impact health-related quality of life (HRQOL). This study examined associations between illness perceptions and cancer-related causal attributions on domains of HRQOL among AYAs with cancer. We also explored differences between demographic and medical characteristics and illness perceptions.

**Methods:**

AYA survivors (*N* = 69; *M*_age_ = 24.1) completed measures of illness perceptions, causal attributions, and HRQOL. Total and item-level scores were calculated for illness perceptions. Causal attributions were coded according to theme (e.g., lifestyle, stress, and genetics). Multiple linear regression models examined associations between illness perceptions and causal attributions on HRQOL. Independent-samples *t*-tests were used to examine differences among demographic and medical characteristics and illness perceptions.

**Results:**

Higher negative levels of illness perceptions were significantly associated with lower HRQOL across all domains. The majority of AYA identified at least one causal attribution, with genetics being the most common. Certain cancer-related causal attributions (i.e., lifestyle, stress, and genetics) were associated with HRQOL. Demographic (i.e., race/ethnicity) and medical characteristics (i.e., treatment status and time since diagnosis) were associated with specific illness perceptions (i.e., identity, treatment control, and consequences).

**Conclusions:**

Findings suggest that holding onto negative illness perceptions is detrimental to HRQOL and highlights specific cancer-related causal attributions that have implications for HRQOL. More research is needed to understand the dynamic changes in HRQOL among AYA survivors over time.

**Implications for Cancer Survivors:**

Understanding AYAs illness perceptions and attributions can help to provide more personalized care and provides talking points for health-care providers aimed at psychoeducation around AYAs cancer diagnosis and trajectory.

## Introduction

Nearly 90,000 adolescents and young adults (AYA; those aged 15–39) are diagnosed with cancer each year in the United States, with > 80% expected to survive beyond 5 years [1]. Given their age and developmental period, this rapidly growing AYA cancer cohort faces several social and emotional challenges [2], as cancer during this time period constitutes a developmentally “off-time” event in their life course [3]. This formative time of significant personal growth and major life milestone achievement is now replaced with focusing on major treatment milestones (e.g., completing chemotherapy), receiving “clean” scans, and returning to work or school [4, 5], while simultaneously grappling with feelings of uncertainty that may accompany recovery from cancer [6, 7]. Coupled with a lower prevalence of cancer during adolescence and young adulthood (~4.2%) [8], the way in which AYAs perceive their cancer, their subjective beliefs (i.e., illness perceptions) and causal attributions may have important implications for HRQOL.

Research on illness perceptions is often guided by the Common-Sense Model (CSM) of Self-Regulation [9]. This model assumes that individuals respond to illness-related symptoms and signs by forming cognitive beliefs (e.g., this illness will last a long/short time) and emotional representations/responses (e.g., I am extremely affected/not at all affected emotionally by this illness) that can influence HRQOL [10, 11]. Illness perceptions can range from negative (e.g., I don’t understand my illness at all) to positive (e.g., I understand my illness very clearly). Threatening or negative illness perceptions have been shown to be associated with detriments to HRQOL among individuals with cancer; however, empirical research examining specific constructs of illness perceptions within the developmentally distinct population of AYAs is limited. The few studies that focus on AYAs with cancer have suggested that more negative illness perceptions are associated with more severe levels of fear of cancer recurrence and higher anxiety and depressive symptoms [12, 13].

In the broader cancer literature, research has suggested that negative illness perceptions in relation to the extent that individuals with cancer experience symptoms, how much cancer affects their lives, and how emotionally affected they are by cancer have been shown to be associated with higher fatigue and psychological distress (anxiety and depressive symptoms) [14, 15]. In addition, research utilizing latent profile analysis to identify illness perception profiles among lung cancer survivors found a “struggling” profile characterized by the largest proportion of patients. This profile captured patients who perceived the most severe consequences from their cancer, the highest symptom severity, the longest expected duration of their cancer, the lowest levels of both perceived personal control over their cancer, and the highest levels of emotional impact. Cancer patients within the struggling profile reported the highest levels of anxiety and depressive symptoms, physical symptoms, pain, and the poorest self-rated health [16].

Parallel to illness perceptions, psychosocial models of disease highlight the importance of causal attributions in influencing HRQOL [17]. Understanding illness-related causal attributions can provide valuable insights into the factors that individuals believe have caused their illness, which is especially important for AYAs with cancer where the root cause of their cancer is often unknown [3, 18]. AYAs may develop theories about the cause of their illness that may or may not be factual. This process is known as attribution theory, where individuals attempt to explain causes of a particular outcome (e.g., cancer) [17, 19]. In the broader cancer literature, specific causal attributions (e.g., environmental exposure, family history, stress, and lifestyle factors) have been found to be associated with lower quality of life [20–22].

Research among causal attributions within AYAs with cancer is limited [23], focused mostly on older adult survivors (≥ 40 years of age) diagnosed during adolescence and young adulthood, on those off active treatment, and solely among survivors of colorectal cancer (CRC). That body of work has suggested that AYA survivors of CRC most often attribute their cancer to heredity, lifestyle, and chance/luck, aligning with causes that are both internal (heredity, lifestyle) and external (chance/luck) as well as causes that are in their control (modifiable; lifestyle) and out of their control (heredity, chance/luck) [23]. AYAs who attributed cancer-related causes as being in their control have been found to be associated with higher levels of anxiety, compared to AYAs who report causes as being out of their control [23]. This is consistent with meta-analyses suggesting that stable (e.g., personality, heredity) and uncontrollable attributions are associated with negative psychological adjustment [19, 24].

Illness perceptions, particularly among younger adults, tend to be stable over time [25], thus representing potentially malleable targets for intervention. Moreover, causal attributions represent unique insights into AYAs’ beliefs about the cause of their illness, which can be a teaching moment for oncology providers to tailor their discussions about the causes of cancer within this growing AYA cancer population. More work is needed among a diverse population of AYA cancer survivors and on the impact of illness perceptions and causal attributions across a variety of health-related quality of life outcomes. Accordingly, the aim of the present study was to understand how AYAs with cancer perceive their illness across cognitive beliefs and emotional representations (i.e., illness perceptions) and to understand AYAs’ beliefs about the cause of their cancer (i.e., causal attributions) to examine how illness perceptions and causal attributions were associated with domains of HRQOL (anxiety, depressive symptoms, fatigue, pain interference, physical function-mobility, social relationships). We also explored differences between demographic and medical characteristics on illness perceptions at the item level. Considering the reviewed literature, it was hypothesized that higher negative illness perceptions and internal causal attributions (e.g., lifestyle and stress) would be associated with lower HRQOL.

## Methods

### Participants and procedures

Eligible AYAs with cancer were recruited in person or over the phone by members of the research team as a part of two larger studies (Study 1: July 2021–May 2022; Study 2: June 2024–October 2024) from two large North-Eastern Hospitals. AYAs were eligible if they were currently an AYA between the ages of 14–39 years and diagnosed with cancer as an AYA (between 14–39) in the past 5 years. While the National Cancer Institute defines AYAs as between the ages of 15–39, given one of the recruitment sites being a Children’s Hospital, and during the COVID-19 pandemic, to increase enrollment efforts, we included adolescents aged 14 in the current study. AYAs were excluded if the oncology team felt that they were not a good fit for the present study (e.g., too sick to participate). Of the 188 AYAs contacted, 14 (7.4%) declined participation and 10 AYAs (18.8%) were lost to follow-up after consent. Following informed consent and assent for AYAs younger than 18, AYAs completed a brief demographic and baseline questionnaire via REDCap. Sixty-nine AYAs completed study participation, resulting in a participation rate of 36.7%. AYAs were compensated with a gift card for completing the questionnaire. All procedures were approved by the appropriate Institutional Review Boards.

### Measures

#### Illness perceptions

Illness perceptions were measured using the Brief Illness Perception Questionnaire (BIPQ)[26] adapted to the situation of cancer. The BIPQ contains 8 items assessing the cognitive and emotional representations of cancer. The first 5 items represent cognitive illness representation (consequences, timeline, personal control, treatment control, identity). The next 2 items represent emotional illness perceptions (concern, emotional response) and the last item assesses illness coherence (understanding). AYAs were asked to rate their agreement with items on a 0–10 scale. Sample items included, “How much does cancer affect your life?’; “How much control do you feel you have over your cancer?” The possible range of scores is 0–80. A total scale score was calculated (including reverse coded scores) with higher scores representing more negative perceptions of cancer. Descriptives and item-level illness perception score analyses did not contain the reverse coded items. Cronbach’s alpha in the present study was 0.67.

#### Causal attributions

Causal attributions were assessed through an open-ended response at the end of the illness perceptions questionnaire, which asked participants to list what they believe are the three most important causal factors of their cancer. Attributions were coded in 7 categories based on prior research [27]. If AYAs’ responses included multiple causal attributions, a separate code was assigned to each reason listed. Reasons other than “I don’t know” and/or no reasons given were condensed into 7 broader categories of causal attributions according to prior research [27]: *Lifestyle* (e.g., alcohol/tobacco, diet, use of hormones, sun exposure, lack of exercise), *Heredity* (e.g., genetics, age, and sex), *Environmental* (e.g., exposure to radiation, microplastics, toxins, occupational hazards, and chemicals), *Prior Health Condition* (e.g., infection, injury), *Psychological* attributes (e.g., mood, weakness, childhood trauma), *Chance/Luck*, and *Stress*. Each causal attribution was also categorized according to locus, internal (e.g., lifestyle) or external (e.g., environmental) to the individual, as well as controllability, in their control, modifiable (e.g., lifestyle) and out of their control, fixed (e.g., heredity and chance/luck).

#### Health-related quality of life

The Patient-Reported Outcomes Measurement Information System Profile Short Form (PROMIS-SF)[28] assessed aspects of health-related quality of life. Either the PROMIS 25 (Pediatric) or PROMIS 29 (Adult) Profile was administered, depending on participant age. These profiles contained a collection of 4-item short forms assessing anxiety, depressive symptoms, fatigue, pain interference, physical function-mobility, and peer/social relationships (i.e., social functioning). Results are reported as *T* scores (mean = 50, standard deviation = 10) with higher scores indicating more of that outcome (e.g., higher depressive symptoms, and higher physical mobility (i.e., function)). Cronbach’s alphas for the sample were as follows: anxiety symptoms (Peds *α* = 0.89, adult *α* = 0.90), depressive symptoms (Peds *α* = 0.94, adult *α* = 0.92), fatigue (Peds *α* = 0.84, adult *α* = 0.93), pain interference (Peds *α* = 0.83, adult *α* = 0.97), physical function-mobility (Peds *α* = 0.83, adult *α* = 0.93), and social functioning (Peds *α* = 0.67, adult *α* = 0.90).

### Data analysis

Descriptive statistics and correlations were conducted for key variables. Associations between demographic (i.e., current age) and medical variables (i.e., time since diagnosis, treatment status, cancer type) were identified a priori and used as covariates in subsequent hypothesis testing, as these variables have been identified in the literature as being associated with HRQOL among AYAs [13, 29]. Multiple linear regression was used to test study hypotheses. In each model, relevant covariates were entered in the first block and the illness perception total score in the second block. We also examined relationships between items on the illness perception scale and HRQOL outcomes. In each model, relevant covariates were entered in the first block and each item on the illness perception scale in the second block. Open-ended responses were categorized by the lead author into lifestyle, genetics, chemical/environmental, prior health condition, psychological, chance/luck, and stress in accordance with prior research, which provides a table for individual attribution responses among individuals with cancer and category coding [27]. Mean differences between the most represented causal attributions and separately locus (internal, external, and both) on HRQOL outcomes were analyzed using ANOVA. Post-hoc analyses for locus were examined using Tukey’s test for multiple comparisons. Independent-samples t-tests were used to examine differences among demographic and medical characteristics and illness perceptions at the item level. Across analyses, the significance level was set at 0.05; analyses were not adjusted for multiple comparisons.

## Results

### Descriptive statistics and identification of covariates

**Fig. 1:**
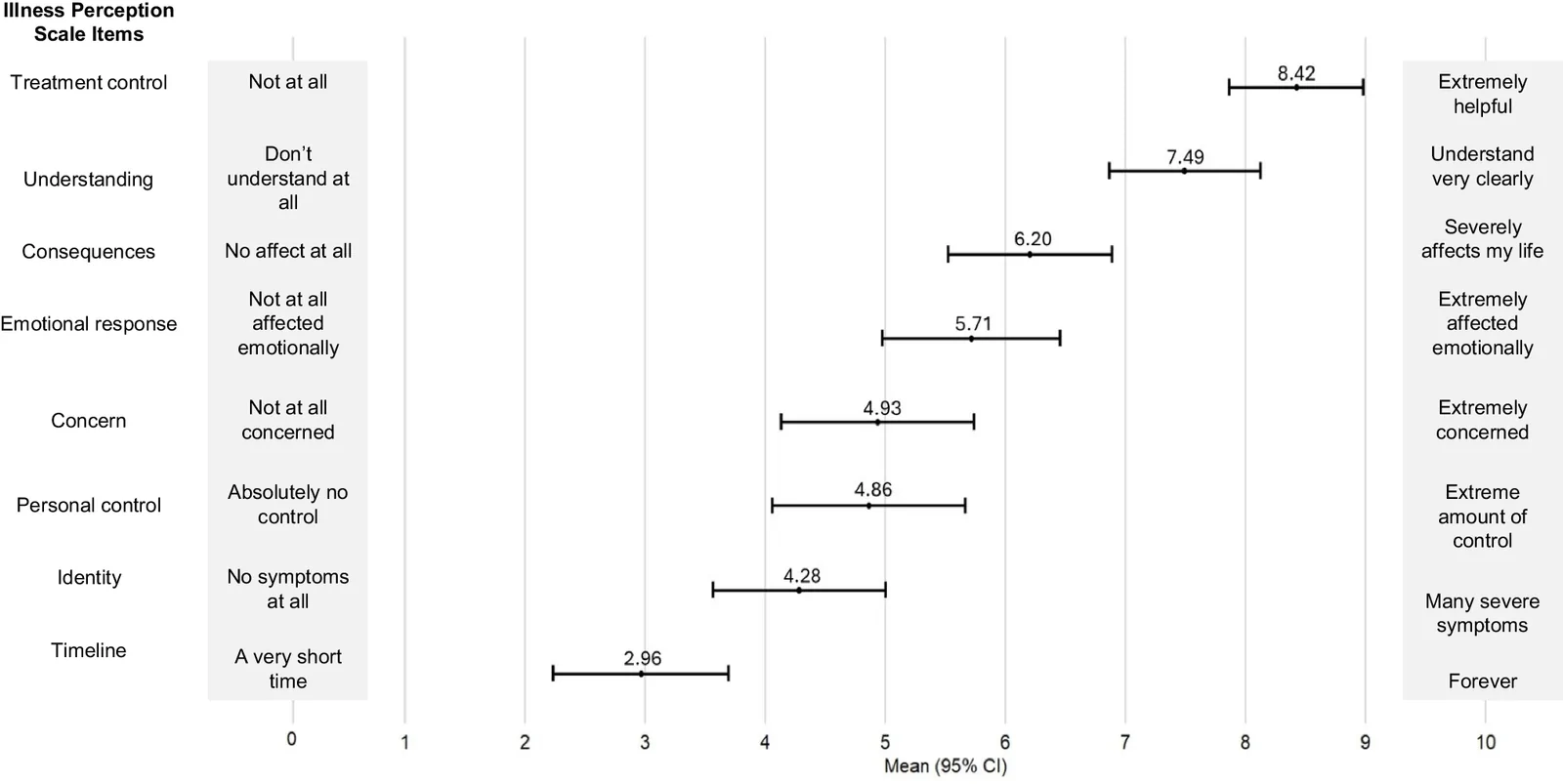
Means and 95% confidence intervals (CIs) of the scale items of illness perceptions

Demographic and medical characteristics of the study sample are displayed in Table 1. AYAs were on average 24 years old (*M* = 24.1, *SD* = 7.2, range = 14–38), were predominately female (55.1%), non-Hispanic white (52.2%), and diagnosed with leukemia/lymphoma (43.5%). AYAs were on average 2.5 years from diagnosis (*M* = 25.4 months, *SD* = 19.7; range = 0.93–66 months) and reported low levels of negative illness perceptions (*M* = 34.55, *SD* = 13.14; range = 3–61). Illness perceptions at the item level ranged from low to high endorsement (see Fig. 1). AYAs reported average levels of HRQOL: anxiety symptoms (*M* = 54.9, *SD* = 9.9), depressive symptoms (*M* = 53.1, *SD* = 10.4), fatigue (*M* = 55.4, *SD* = 11.0), pain interference (*M* = 49.0, *SD* = 9.6), physical function-mobility (*M* = 47.5, *SD* = 10.6), and peer/social relationships (*M* = 49.8, *SD* = 9.7). Across the two different sites, there were significant differences in HRQOL with respect to physical functioning-mobility and anxiety symptoms, with AYAs recruited from the Children’s Hospital experiencing lower physical functioning (*M* = 39.2, *SD* = 11.0) and anxiety symptoms (*M* = 49.7, *SD* = 9.7) compared to AYAs recruited at a comprehensive cancer center not devoted solely to children (*M* = 50.6, *SD* = 8.7; *M* = 56.8, *SD* = 9.3, respectively). Causal attributions and exemplary causes reported by AYAs are presented in Table 2. Most AYAs with cancer (60.9%) identified one or more cancer-related causal attributions. Collectively, AYAs attributed most causes of cancer to genetic/heredity factors (37.7%), followed by lifestyle factors (36.2%), chemical/environmental factors (31.9%), chance (20.9%), stress (21.7%), prior health condition or health-related event (13.0%), and psychological factors (9.7%). Approximately 20% of AYAs (*n* = 12, 17.4%) did not report any cause related to their cancer, either stating that they did not know or that nothing had caused their cancer.

**Table 1:** Demographic and medical characteristics of the study sample (*N* = 69)

Variable	*n* (%)	Range
Age, mean (SD)	24.1 (7.2)	14–38
Female	38 (55.1%)	
Race/ethnicity		
Non-Hispanic White	36 (52.2%)	
Non-Hispanic Black/African American	9 (13.0%)	
Non-Hispanic Asian	5 (7.2%)	
Hispanic/Latino	16 (23.2%)	
Non-Hispanic Other/none selected	3 (4.3%)	
Cancer type		
Leukemia/lymphoma	30 (43.5%)	
Thyroid	9 (13.0%)	
Breast	6 (8.7%)	
Testicular	6 (8.7%)	
Other^a^ (< 5 AYA diagnoses)	18 (26.1%)	
Treatment status		
Active treatment	39 (56.5%)	
Completed treatment	30 (43.5%)	
Months since diagnosis, mean (SD)	25.4 (19.7)	0.93–66 months

^a^Colon, sarcoma, pancreatic, gynecological, brain, small intestine, melanoma

**Table 2:** Open-ended causal attributions

Causal attribution	Endorsement [*n *(%)]	Individual attribution reasons
Genetic/heredity *Internal, out of AYA’s control*	26 (37.7%)	Family history; lynch syndrome; I had a 0.4% chance to get it, and I did; genes; unlucky genetics
Lifestyle *Internal, in AYA’s control*	25 (36.2%)	Daily unhealthy food intake; alcohol; sun exposure; not using the bathroom; life
Chemical/environmental *External, out of AYA’s control*	22 (31.9%)	Exposure to chemicals; home/work environment; food ingredients; clothes/gear; pollution
Chance *External, out of AYA’s control*	20 (20.9%)	Bad luck; the devil; random event; fate Just happened to be an unlucky one
Stress *Internal, in AYA’s control*	15 (21.7%)	Personal stress; familial stress; school stress; emotional stress
Prior health condition or health-related event *External, out of AYA’s control*	9 (13.0%)	Virus; relapse; too much sickness; blood cells
Psychological *Internal, out of AYA’s control*	6 (8.7%)	Childhood trauma; depression; anxiety
I don’t know/nothing	12 (17.4%)	–

At the item level, we examined differences between demographic [age cohort (14–19 (*n* = 20), 20–29 (*n* = 29), 30–39 (*n* = 20), sex, race/ethnicity (non-Hispanic white vs. different races)] and medical factors [treatment status (on vs. off treatment), time since diagnosis (0–12 months vs. more than 1 year), cancer type (leukemia/lymphoma vs. other)] on illness perceptions at the item level. AYAs identifying as other than non-Hispanic white (i.e., Black, Asian, Native Hawaiian/Pacific Islander, Hispanic) were more likely to endorse experiencing symptoms (e.g., pain and fatigue) (*t* = -2.5, *p* = 0.01) from their cancer (*M* = 5.2, *SD* = 2.8) compared to AYAs identifying as non-Hispanic white (*M* = 3.4, *SD* = 3.0), and less likely to feel that their cancer-related treatment can help their cancer (*t* = 2.1, *p* = 0.04; *M* = 7.8, *SD* = 2.6 vs. *M* = 9.0, *SD* = 2.0, respectively). No demographic differences were found for age cohort and sex. AYAs diagnosed within the past 12 months were more likely to report that cancer more severely affects their life (*M* = 7.3, *SD* = 2.2) compared to AYAs diagnosed more than 1 year ago (*M* = 5.6, *SD* = 3.1). AYAs on active cancer treatment were also more likely to report that cancer more severely affects their life (*M* = 6.9, *SD* = 2.6) and that they experience more cancer-related symptoms (*M* = 4.9, *SD* = 2.7) compared to AYAs who completed cancer treatment (*M* = 5.3, *SD* = 3.1; *M* = 3.4, *SD* = 3.2, respectively). There were no differences among cancer type and illness perception scores at the item level.

### Hypothesis testing

Results of the regression analyses are presented in Table 3. Controlling for significant demographic (age; continuous) and medical characteristics [time since diagnosis (continuous), cancer type (leukemia/lymphoma vs. other cancers), treatment status (on vs. off active treatment)], illness perceptions were negatively associated with physical (*β* = −0.26, *p* = 0.02) and social (*β* = −0.52, *p* < 0.001) functioning and positively associated with anxiety symptoms (*β* = 0.39, *p* < 0.001), depressive symptoms (*β* = 0.34, *p* = 0.002), fatigue (*β* = 0.27, *p* = 0.02), and pain (*β* = 0.34, *p* = 0.004). Focusing on item-level analyses, controlling for the same covariates in the previous models, higher levels of identity (symptoms) were negatively associated with physical functioning (*β* = -0.38, p = 0.01) and positively associated with fatigue (*β* = 0.51, *p* = 0.002) and pain (*β* = 0.49, *p* = 0.002); higher levels of consequences (how much cancer affects your life) were negatively associated with both anxiety (*β* = −0.55, *p* < 0.001) and depressive symptoms (*β* = −0.34, *p* = 0.04); and higher levels of emotional response (how much cancer affects you emotionally) were positively associated with both anxiety (*β* = 0.74, *p* < 0.001) and depressive symptoms (*β* = 0.57, *p* < 0.001) and negatively associated with social functioning (*β* = −0.46, *p* = 0.01).

**Table 3:** Associations between illness perceptions and health-related quality of life outcomes

Dependent variables	Model	Independent variables	*B*	*SE*	*β*	*p*	95% Confidence Interval	
							**Lower**	**Upper**
Physical functioning	1	Age	0.37	0.18	0.25	0.04	0.01	0.73
		Time since dx	0.12	0.07	0.23	0.08	−0.01	0.26
		Cancer type	−1.43	2.41	−0.07	0.56	−6.24	3.38
		Treatment status	3.36	2.64	0.16	0.21	−1.81	8.64
		Illness perceptions	−0.21	0.09	−0.26	0.02	−0.38	−0.04
		Overall model: *F*(5,68) = 5.8, *p* < 0.001; Adj. *R*^2^ = 0.3						
Social functioning	2	Age	0.12	0.17	0.09	0.48	−0.21	0.45
		Time since dx	−0.06	0.06	−0.12	0.35	−0.19	0.07
		Cancer type	−2.33	2.23	−0.12	0.30	−6.79	2.11
		Treatment status	3.71	2.44	0.19	0.13	−1.17	8.59
		Illness perceptions	−0.38	0.08	−0.52	< 0.001	−0.54	−0.22
		Overall model: *F*(5,68) = 5.6, *p* < 0.001; Adj. *R*^2^ = 0.3						
Anxiety symptoms	3	Age	0.39	0.16	0.28	0.02	0.07	0.70
		Time since dx	0.12	0.06	0.24	0.05	−0.001	0.24
		Cancer type	5.30	2.11	0.27	0.01	1.10	9.52
		Treatment status	−4.49	2.31	−0.23	0.06	−9.10	0.13
		Illness perceptions	0.29	0.08	0.39	< 0.001	0.14	0.44
		Overall model: *F*(5,68) = 8.3, *p* < 0.001; Adj. *R*^2^ = 0.4						
Depressive symptoms	4	Age	0.52	0.18	0.36	0.01	0.17	0.87
		Time since dx	0.04	0.07	0.07	0.57	−0.10	0.17
		Cancer type	3.96	2.35	0.19	0.10	−0.73	8.65
		Treatment status	−3.89	2.58	−0.19	0.14	−9.03	1.26
		Illness perceptions	0.27	0.08	0.34	0.002	0.10	0.43
		Overall model: *F*(5,68) = 6.1, *p* < 0.001; Adj. *R*^2^ = 0.3						
Fatigue	5	Age	−0.12	0.21	−0.08	0.57	−0.53	0.30
		Time since dx	0.02	0.08	0.03	0.81	−0.14	0.18
		Cancer type	3.96	2.77	0.18	0.16	−1.58	9.49
		Treatment status	−5.48	3.04	−0.25	0.08	−11.54	0.59
		Illness perceptions	−0.23	0.10	0.27	0.02	0.03	0.42
		Overall model: *F*(5,68) = 2.3, *p* < 0.05; Adj. *R*^2^ = 0.1						
Pain	6	Age	−0.08	0.18	−0.06	0.67	−0.43	0.28
		Time since dx	−0.05	0.07	−0.11	0.43	−0.19	0.08
		Cancer type	4.65	2.35	0.24	0.05	−0.05	9.34
		Treatment status	−2.20	2.58	−0.11	0.40	−7.34	2.95
		Illness perceptions	0.25	0.08	0.34	0.004	0.09	0.42
		Overall model: *F*(5,68) = 3.2,* p* < 0.05; Adj. *R*^2^ = 0.1						

Mean differences between the most frequently mentioned causal attributions and HRQOL outcomes are presented in Table 4. There was a significant mean difference in depression (*F* = 4.2, *p* = 0.04) with higher levels of depressive symptoms among AYAs who attributed their cancer to lifestyle factors. Higher levels of both anxiety (*F* = 6.7, *p* = 0.01) and depressive symptoms (*F* = 6.0, *p* = 0.02) were also seen among AYAs who attributed the causes of their cancer to stress.

**Table 4:** Mean differences between health-related quality of life outcomes and top five mentioned causal factors

Causal factors	Physical functioning	Social functioning	Anxiety symptoms	Depressive symptoms	Fatigue	Pain
Genetic	*F* = 7.8, *p* = 0.01	–	*–*	*–*	–	–
Yes (*n* = 26)	51.80 (8.42)	48.87 (10.74)	56.59 (8.38)	54.17 (8.86)	55.56 (11.61)	47.91 (9.23)
No (*n* = 43)	44.81 (10.97)	50.34 (9.16)	53.80 (10.63)	52.39 (11.27)	55.24 (10.69)	49.61 (9.83)
Lifestyle	–	–	*–*	*F* = 4.2, *p* = 0.04	–	–
Yes (*n* = 25)	48.88 (9.52)	48.07 (9.21)	57.42 (8.4)	56.40 (10.05)	55.83 (9.98)	51.57 (9.42)
No (*n* = 44)	46.64 (11.17)	50.76 (9.99)	53.39 (10.27)	51.17 (10.22)	55.09 (11.58)	47.49 (9.45)
Chemical/Environmental	–	–	–	–	–	–
Yes (*n* = 22)	47.85 (9.96)	48.55 (8.27)	57.20 (8.63)	54.11 (11.09)	56.70 (9.78)	49.55 (10.84)
No (*n* = 47)	47.27 (10.96)	50.36 (10.38)	53.75 (10.31)	52.57 (10.14)	54.74 (11.52)	48.69 (9.03)
Chance	–	–	–	–	–	–
Yes (*n* = 20)	46.36 (11.58)	49.32 (10.28)	51.78 (11.47)	51.53 (11.37)	54.62 (10.80)	48.26 (9.67)
No (*n* = 49)	47.90 (10.24)	49.98 (9.60)	56.10 (8.97)	53.69 (10.03)	55.66 (11.12)	49.26 (9.61)
Stress	–	–	*F* = 6.7, *p* = 0.01	*F* = 6.0, *p* = 0.02	–	–
Yes (*n* = 15)	47.67 (10.08)	48.30 (7.20)	60.46 (5.26)	58.67 (10.53)	56.79 (8.63)	50.91 (8.98)
No (*n* = 54)	47.39 (10.81)	50.20 (10.34)	53.29 (10.31)	51.51 (9.90)	54.96 (11.56)	48.43 (9.57)

– not significant

Lastly, there was a significant mean difference in physical functioning (*F* = 7.8, *p* = 0.01) with higher levels of physical functioning among AYAs who attributed their cancer to genetic/family history causes. There was a significant mean difference in anxiety symptoms among attributions coded according to locus (internal or external). Post-hoc analyses found that AYAs who solely attributed cancer-related causes as being internal (e.g., lifestyle) (*n* = 19) had significantly higher anxiety symptoms (*M* = 58.2, *SD* = 8.5) compared to AYAs (*n* = 10) who solely attributed cancer-related causes as being external (e.g., environmental) (*M* = 48.9, *SD* = 11.0). Cell sizes were too small to examine mean differences among AYAs who solely attributed cancer-related causes to aspects of controllability (modifiable or fixed).

## Discussion

The aim of this current study was to examine the role of illness perceptions and causal attributions on health-related quality of life among AYAs with cancer. Overall, our results suggest that higher negative illness perceptions among AYAs were associated with lower HRQOL across all domains (physical, social, anxiety, depression, fatigue, pain). In addition, several illness perception items were associated with varying aspects of HRQOL. We also found that AYAs were most likely to attribute causes of their cancer to factors associated with genetics/heredity, lifestyle, chemical/environmental, chance, and stress. AYAs solely holding onto internal attributions, such as lifestyle factors and stress, were more likely to exhibit higher levels of anxiety. Our findings extend previous research examining illness perceptions and causal attributions among AYAs with cancer by providing insight into specific cognitive and emotional representations of cancer that have implications for HRQOL. Results confirm the relevance of the Common-Sense Model of Self-Regulation and attribution theory for describing illness perceptions and cancer-related causal attributions and point towards targets for intervention that can address psychoeducational needs for AYAs with cancer.

Focusing on illness perceptions, AYAs on average rated moderate levels of symptoms (identity), concern, and personal control over their cancer, with higher levels seen among emotional response and consequences. Notably, AYAs seem to perceive their cancer as temporary and not lasting a long time (timeline). While the majority of AYAs (65.2%) were diagnosed with cancer more than 1 year ago, we found no significant differences between timeline and time since diagnosis, suggesting that while AYAs are often aware that cancer is considered a chronic disease, it will not last forever. This is consistent with previous research focusing on thyroid cancer survivors that, compared to middle- and older-aged adults, AYAs showed less negative perceptions of their illness continuing into the future [13]. However, while AYAs may not perceive their cancer to last a long time, AYA cancer survivors face several long-term medical and psychosocial late effects from cancer (e.g., cardiotoxicity, secondary cancers, cognitive ability, and relationship changes). The completion of cancer-related treatment may not constitute an immediate return to their pre-cancer lives [30] and requires significant long-term follow-up care focused on survivorship [31]. While this is often a topic for discussion among AYAs, framing conversations around the treatment aspect of cancer potentially not lasting a long time within the context of long-term survivorship may be beneficial for AYAs. Perceptions of timeline may also have been influenced by perceptions of treatment control. On average, AYAs felt that treatment for their cancer was/is extremely helpful.

Higher scores on illness perception items relating to symptoms and treatment control were associated with demographic variables in that AYAs identifying as other than non-Hispanic white (i.e., Black, Asian, Native Hawaiian/Pacific Islander, Hispanic) were more likely to endorse experiencing more symptoms (e.g., pain, fatigue) from their cancer and less likely to perceive their cancer-related treatment to be helpful. This is significant as a large body of research demonstrates racial and ethnic disparities in cancer treatment, cancer symptoms (e.g., pain), and cancer outcomes [32–35]. Increased awareness of these disparities among AYAs with cancer is essential to mitigate differences in treatment and to identify barriers to care. In addition, higher scores on illness perception items of symptoms and consequences were associated with medical variables in that AYAs diagnosed within the past 12 months as well as AYAs on active cancer treatment were more likely to perceive that cancer more severely affects their life. In addition, AYAs on active cancer treatment were also more likely to perceive more cancer-related symptoms. Together, these findings point to specific subgroups within AYAs with cancer that may benefit from more targeted and ongoing support during diagnosis and treatment and into survivorship.

Surprisingly, we found that AYAs perceived they understood their cancer very clearly. Given that 60.9% of AYAs identified a causal attribution for their cancer, it is likely that they felt they understood the etiology of cancer but not the specific cause of their cancer. Our causal attribution findings suggest that AYAs tend to ascribe causes of their cancer to both internal and external factors. While some causal attributions that AYAs mentioned are more likely to be rooted in the actual cause of their cancer, such as genetics and family history, other AYAs attributed causal attributions may be less likely to be rooted in the cause of the cancer, such as psychosocial stressors and certain environmental exposures. Our results additionally produce common themes of an external locus of control among AYAs (and cancer fate being at others’ discretion), which highlights an area for intervention, as multiple studies have found associations between external locus of control and decreased HRQOL, psychological well-being, and coping skills among individuals with cancer [36–38]. Overall, given that AYAs often noted multiple causal attributions for their cancer, future research should consider a focus on psychoeducation targeted towards perceived causal attributions surrounding AYAs’ cancer diagnoses.

When examining specific causal attributions of cancer, we found that AYAs most frequently attributed the cause of their cancer to genetic/heritability factors (37.7%). Genetics often play a role in cancer etiology among AYAs; however, lifestyle can exacerbate genetic predisposition [39]. Interestingly, within our sample, AYAs who attributed their cancer to genetic causes also had significantly higher levels of physical functioning. It is probable that those with genetic predisposition had an early awareness of increased cancer risk and have therefore made healthy choices throughout their life to avoid unfavorable outcomes that they may have experienced vicariously through family members with the same genetic cancer [40]. This may have served as a teachable moment among AYAs: a time after a serious health event that increases the likelihood of improving health behaviors in efforts to prevent disease [41].

AYAs also attributed their cancer almost equally to lifestyle factors (36.2%) as to genetics. While many causal attributions denoted an external locus of control, this suggests that AYAs with cancer may place internal blame for engaging in health-compromising behaviors (e.g., smoking, alcohol use, and unhealthy diet) leading to their diagnosis. While approximately 40% of cancer is attributable to potentially modifiable factors [42], this tends to occur over the course of one’s lifetime [43, 44] and may not be directly associated with cancer among AYAs. We also found depressive symptoms to be significantly higher among AYAs with lifestyle attributions. These findings may represent regret, guilt, and/or shame among AYAs who attribute their modifiable behaviors to their cancer. Therefore, internal causal attributions may require targeted supports to protect against negative psychological symptoms (depression, anxiety). A growing body of research has suggested that individuals who believe their cancer was not caused by their own actions or behaviors tend to cope better and experience fewer negative feelings [45].

Other cancer-related causal attributions draw attention to a multitude of other factors that AYAs perceive to be responsible for their cancer, including chemical and environmental exposures, which can be uncontrollable especially for individuals exposed to certain environments through work and life. AYAs also mentioned cancer-related causes aligning with chance, presumably in the control of a larger entity/force (e.g., “Bad luck,” “The Devil,” and “Fate”), aligning with previous research [13]. While we did not find significant associations between chance attribution and HRQOL, other research has suggested that attributing cancer to bad luck/chance is associated with better quality of life among childhood cancer survivors and lower anxiety among AYAs with cancer [22, 23].

Lastly, stress-related attributions were endorsed by approximately a quarter of AYAs and were significantly associated with both higher depressive and anxiety symptoms. Chronic stress can interfere with individuals’ ability to cope and adjust to their illness, potentially undermining the ability to adapt to and control their stress, possibly intensifying psychological distress [46]. While stress in general is difficult to tease apart, the context in which stress is noted qualitatively within our findings (e.g., personal stress, familial stress, school stress, and emotional stress) might provide insight into the types of stressors most prominent in AYAs’ day-to-day lives that are perceived to be attributable to their cancer. Further understanding these specific stressors may inform stress management and coping techniques specific to AYAs and promote a sense of control over cancer-related causes to reduce psychological burden.

This study has several strengths, including a comprehensive examination of illness perceptions and causal attributions on several domains of HRQOL among a diverse demographic and clinical sample of AYAs diagnosed with cancer. However, it is not without limitations. The smaller sample size and results not adjusted for multiple comparisons warrant caution in the interpretation of results. The current study was also cross-sectional in nature, precluding the ability to look at changes in perceptions and attributions over time. While research has suggested that illness perceptions tend to be stable [25], future research would benefit from examining relationships over time, across the survivorship continuum and at differing stages of diagnosis and treatment (e.g., early stage vs. metastatic and surgery only), as these relationships may differ.

In addition, while the results of our study support relationships between illness perception and causal attributions on HRQOL, the possibility of bidirectional relationships or alternate explanations remains. For example, domains of HRQOL could influence how AYAs perceive their illness identity (i.e., heighten how they experience their symptoms) and/or how they influence AYAs’ cancer-related causal attributions (e.g., depressive symptoms leading individuals to believe they were the cause of their cancer). Similarly, relationships between HRQOL, illness perceptions, and causal attributions are likely to be more dynamic and reciprocal than the unidirectional approach suggested by our statistical analyses. To further support these findings, future research would benefit from longitudinal designs and ecological momentary assessment methods to provide a more comprehensive understanding of these relationships as they unfold over time.

## Conclusion

This study highlights the importance of considering the impact of illness perceptions and cancer-related causal attributions on HRQOL among AYAs with cancer. Largely, our results suggest that negative illness perceptions were associated with worse HRQOL, less controllable attributions were associated with higher anxiety, and controllable/modifiable attributions were associated with depressive and/or anxiety symptoms. We suggest a need to address these cognitive and emotional representations and attributions of cancer within the AYA cancer population to promote HRQOL. Interventions focused on aspects of cognitive behavioral therapy and coping skills training may allow AYAs to reframe their negative illness perceptions and introduce a sense of control. Additionally, it may be particularly helpful for oncology providers to address causal attributions near diagnosis, which may impart a better sense of understanding among AYAs and may be particularly beneficial for AYAs holding onto cancer-related attributions, which they perceive to have caused or be brought upon them by some external force (e.g., stress and fate). Similarly, introducing positive lifestyle changes and stress management techniques for AYAs with cancer may serve to boost coping strategies and limit the negative influence of causal attributions. Future work in this area should consider these approaches when designing interventions to address the negative impacts of illness perceptions and causal attributions on HRQOL.

## Data Availability

The datasets generated and analyzed during the current study are available for researchers at a responsible request from the first author.
